# Workshop—Predicting the Structure of Biological Molecules

**DOI:** 10.1002/cfg.414

**Published:** 2004

**Authors:** Damian Counsell

**Affiliations:** Rosalind Franklin Centre for Genomics Research Wellcome Trust Genome Campus Cambridge CB10 1SB UK

## Abstract

This April, in Cambridge (UK), principal investigators from the Mathematical Biology Group of the Medical Research Council's National Institute of Medical Research organized a workshop in structural bioinformatics at the Centre for Mathematical Sciences. Bioinformatics researchers of several nationalities from labs around the country presented and discussed their computational work in biomolecular structure prediction and analysis, and in protein evolution. The meeting was intensive and lively and gave attendees an overview of the healthy state of protein bioinformatics in the UK.


**Comparative and Functional Genomics**
*Comp Funct Genom* 2004; **5**: 480–490.
Published online in Wiley InterScience (www.interscience.wiley.com). **DOI:** 10.1002/cfg.414

**Feature**

### Workshop — Predicting the Structure of Biological Molecules

### Isaac Newton Institute, Centre for Mathematical Sciences, Cambridge University, UK, 26–30 April 2004

Damian Counsell*
*Rosalind Franklin Centre for Genomics Research, Wellcome Trust Genome Campus, Cambridge CB10 1SB, UK*

**Correspondence to: Damian Counsell, RFCGR, Wellcome Trust Genome Campus, Cambridge, CB10 1SB, UK. E-mail: d.counsell@rfcgr.mrc.ac.uk*

Received: 10 June 2004
Revised: 18 June 2004
Accepted: 21 June 2004

### Abstract

**This April, in Cambridge (UK), principal investigators from the Mathematical Biology Group of the Medical Research Council's National Institute of Medical Research organized a workshop in structural bioinformatics at the Centre for Mathematical Sciences. Bioinformatics researchers of several nationalities from labs around the country presented and discussed their computational work in biomolecular structure prediction and analysis, and in protein evolution. The meeting was intensive and lively and gave attendees an overview of the healthy state of protein bioinformatics in the UK. Copyright © 2004 John Wiley & Sons, Ltd.**

**Keywords:** **protein bioinformatics; structural genomics; protein structure prediction; molecular evolution; molecular simulation; structural bioinformatics**

This workshop was organized by members of the Computational Biology Group at the UK Medical Research Council's National Institute for Medical Research (**http://mb2.nimr.mrc.ac.uk/**), Franca Fraternali, Richard Goldstein and Willie Taylor. It was a low-key affair, organized late, yet it was probably the best scientific meeting I have ever attended; I was interested in advance in the content of practically every session. Most of the seminars were well-prepared, clear, relevant and refreshingly concise. Even allowing for usually well-informed questions and interruptions, sessions rarely overran (or if they did, it didn't feel that way). Unfortunately, because I heard about the meeting only shortly before it took place, I was unable to attend every presentation in full. Although the speakers and attendees were of many nationalities, they are all currently working in the UK.

After Willie Taylor's introduction it was appropriate that **Cyrus Chothia** (Laboratory of Molecular Biology, Cambridge, UK; **http://www.mrc-lmb.cam.ac.uk/genomes/Cyrus.html**), one of the most prominent and longstanding researchers in the field of protein structure bioinformatics in Cambridge, should open proceedings. In his talk, 'Structural constraints on protein mutations', he described his work with Rajkumar Sasidharan (**http://www.mrc-lmb.cam.ac.uk/genomes/sraj/**). It was good for the rest of the meeting that, regardless of Chothia's standing, there was no reluctance to challenge his arguments and his contribution provoked the first of many stimulating discussions that took place both during and after presentations.

When questioned, Chothia admitted to an intentional looseness with the term 'positive selection' in his description of the degree and type of residue type conservation in different locations in protein structures. He outlined how residue conservation varied with degree of site exposure and summarized the residue properties most likely to be shared in the same sites across homologues. Among the intriguing statistics he presented, Chothia noted that the normalized frequency of changes in surface residues was five to six times higher than core residues. The most 'selected' (conserved) residue positions were least likely to vary in their size first, followed by their physicochemical character.

Copyright © 2004 John Wiley & Sons, Ltd.

For the least 'selected' positions, the priorities were reversed.

In summary: average selectivity values for given sites in proteins are calculable, the frequency of variation can be explained in terms of the properties and locations of the analysed sites, and the frequency with which residues vary at given sites had a medium correlation with the overall underlying frequency of random mutations. Richard Goldstein asked if Sasidharan and Chothia's study showed that proteins tended towards robustness and Chothia admitted not. Willie Taylor asked about possible resemblances between the substitution matrix derived from Chothia's structure-based alignments and the Dayhoff matrix. Chothia said that the two were similar.

**Juan Fernandez-Recio** (Crystallography and Biocomputing Unit, Department of Biochemistry, University of Cambridge; **http://www-cryst.bioc.cam.ac.uk/~juan/**) next presented 'Protein–protein docking by global energy minimization', work that he began in Ruben Abagyan's lab at the Scripps Institute [9]. He aims to find general methods for predicting the structures of protein–protein complexes based solely on the structures of the members of those complexes. Useful because structures of complexes are hard to determine, these have increased in importance as lower-resolution structure determination methods have become more powerful and generated more data.

While cheaper analyses treating docking partners as rigid bodies are easier to calculate, they produce unrealistic energy landscapes, unlikely to lead to even approximately correct solutions. Models including fully flexible protein structure require the exploration of huge conformational spaces. Fernandez-Recio and co-workers seek a compromise: the first step is to treat structures as rigid bodies with 'soft' van der Waals' radii permitting atomic overlap; the second step is to permit flexibility elsewhere. Other efficiencies come through representing molecules in terms of their internal, rather than Cartesian, coordinates. This combination resulted in one of the top 'blind' performers in the (CASP-like) CAPRI protein docking prediction competition (**http://capri.ebi.ac.uk/**). Unlike many reviews of bioinformatics methods by their developers, Fernandez-Recio went on to give examples of both the successful and unsuccessful applications of his approach. He also discussed some of the other uses for the output of his docking simulations — they can be used, for example, in the prediction of binding patches on proteins.

Everyone I spoke to was especially impressed with the volume and depth of analysis that had been performed by **Sanne Abeln** (**http://www.stats.ox.ac.uk/people/students.htm**), still a first-year student in Charlotte Deane's bioinformatics group in the Statistics Department at Oxford University. In 'Fold usage on genomes and protein structure evolution' she described her huge survey of protein structures across species. She compared the number of distinct folds with genome size, examined the number of occurrences of folds, 'duplications' of folds, and families per fold and related them. She had asked what these data could say about the 'ages' of folds, evolutionary mechanisms and evolutionary relationships between folds. By taking large sequence sets (150 + genomes from all kingdoms) and widely used bioinformatics tools (PSI-BLAST and SCOP), and applying them on a large scale, she not only made too many interesting observations to list here, but had already begun to devise plausible explanations for many of the phenomena she observed.

It seems that distributions of the popularity of folds are often described by power laws. Some folds at least appear to be missing in certain genomes. The data she collected for $\alpha\beta$ proteins are different from folds in the other fold classes (similar comparisons against $\alpha\beta$ proteins were made at several points over the course of the meeting). Abeln cautioned that it is very difficult to make phylogenetic trees from this kind of data since:

- There are no clear relations between the different measures of fold usage (i.e. occurrences of folds across genomes, duplications of folds on a genome, and families per fold).
- When a fold diverges to a new fold on one genome, occurrence and duplications are set back to one, and it is therefore difficult to obtain evolutionary relations between folds from these measures.

Interesting power law-based relations also emerged from their analyses of fold distributions across families and superfamilies. Just as there had been discussion of Chothia's use of the term 'positive selection', there was some debate over Abeln's allusions to 'old folds' in her discussion of the

Copyright © 2004 John Wiley & Sons, Ltd.

possible evolution of folds. The idea of 'trapped folds' having difficulty evolving was another theme which re-emerged later in the week, when Ben Blackburne described his hugely simplified *in silico* minimal proteins.

The second day was chaired by Richard Goldstein and the first speaker, **Kenji Mizuguchi** (Department of Biochemistry, University of Cambridge, UK, **http://www-cryst.bioc.cam.ac.uk/~kenji/**), addressed **'**Sequence–structure homology recognition**'**. Mizuguchi first clearly described the central problems of homology modelling: identifying the best structural templates against which to model the sequence of an unknown fold and finding the best alignment between that sequence and its target. He was classically biological in his use of terminology, distinguishing between the identification of analogous (corresponding, but not related) and truly homologous (corresponding and related) folds.

After an overview of existing methods for fold recognition and alignment, he outlined FUGUE (**http://www-cryst.bioc.cam.ac.uk/fugue/**), a system he developed along with Jiye Shi and Tom Blundell [18]. FUGUE exploits structural data in the form of environment-specific substitution tables — 64 of them — and gap penalties. These are applied alongside modern sequence alignment techniques and refined by testing to see how the environment definitions affect performance. Mizuguchi claimed 70–100 hits/day on the FUGUE Website and that the method outperforms other blind prediction servers in alignment/assignment. Unfortunately, Mizuguchi's clear explanation of the problems and approach didn't leave him time to discuss other applications, but I look forward to reading about them elsewhere [19,20,22]. It was also satisfying during questioning afterwards to hear him be sensibly dismissive of any attempt to attach statistical confidence values to FUGUE's output, given the absence of an underlying mathematical model. For a wider view of the importance of fold recognition, he recommended his review in *Drug Discovery Today* [17].

**Franca Fraternali** (**http://mathbio.nimr.mrc.ac.uk/taylor/members/ffranca/**) was the first of the organizers to lead a seminar. She described the parametrization of a simple and easy-to-derive analytical formula for taking account of solvent effects in molecular dynamics simulations, using accessible surface areas. The method, parametrically optimised surfaces (POPS) [10], has already been integrated into GROMOS96, and demonstrated to be only about 30% slower than vacuum methods — orders of magnitude cheaper than explicit water molecular dynamic simulations.

In order to obtain an energy term to add the solvent contribution to the force field, one needs to have solvation parameters that, multiplied with the surface terms, give the free energy of solvation. So far, theoreticians have used experimentally-obtained solvation energies of transfer of atoms from water to vapour. Fraternali sketched out a new approach to the calculation of these parameters that makes use of explicit water simulations on a selected number of conformations of different peptides and proteins. From solute-restrained MD simulations of these conformers, calculated in explicit water, it is possible to obtain distributions of the atomic forces exerted by that water and thereby parametrize the POPS forces accordingly.

For the second part of her talk, Fraternali concentrated on more bioinformatic analysis of structural data using POPS. The method has been parametrized in order to reproduce solvent accessibilities at atomic level (POPSA) and at the residue level (POPSR), based on a training set of about 100 proteins of different sizes and topologies. The formula reproduces accessibilities calculated with the program NACESS with less than 10% error.

Fraternali has shown how the formula proved useful in identifying protein–protein and protein–RNA interactions in large macromolecular assemblies like the ribosome — even based on low resolution structures (C-$\alpha$ and P atoms only) like the 70S ribosome. Differences between the 30S as a separate subunit and as part of the 70S complex (with the 50S subunit) have been highlighted in this way. Because of the presence of the P-tRNA in the 70S ribosome, localized conformational rearrangements occurring within the subunits, exposing Arg and Lys residues to negatively charged binding sites of P-tRNA, can be identified. POPSR can also be used to estimate the loss of free energy of solvation upon complex formation, particularly useful in designing new protein–RNA complexes and in suggesting more focused experimental work.

Like many of the most effective bioinformatic approaches, POPS is an approximation to make large-scale problems tractable. In this case, Fraternali used it to tackle the problem of the large multi-component ribosome structures and to produce

Copyright © 2004 John Wiley & Sons, Ltd. 

illuminating data. A POPS web server has been made available at **http://ibivu.cs.vu.nl/programs/popswww/** [6].

**Michele Vendruscolo**'s (**http://www.ch.cam.ac.uk/CUCL/staff/mv.html**) group, in Cambridge's Chemistry Department, studies non-native structures of proteins and uses molecular dynamics to translate experimental measurements into structures. Vendruscolo made the important point that we know far less about the cellular states of proteins than about their crystal states, as determined by X-ray crystallography. We urgently need to understand the forms proteins take when they form aggregates, intermediates, assemblies, or when they are the nuclei of misfolded forms.

Vendruscolo outlined his group's use of *restrained simulations* to investigate such problems. The approach generates an ensemble of structures for study for which specific experimentally-measured restraints are satisfied. Various experimental techniques can be used to obtain the restraints. Vendruscolo outlined the technique with an example of three amino acids for which a dozen or so interactions and specific bonds had to be satisfied. Once an experimental technique and a structural interpretation of the derived data have been chosen, the model for the interactions emerges and a pseudo-energy function penalizes deviations from the experimentally derived restraints. Vendruscolo argued that these were essential because molecular dynamics simulations cannot entirely replace experiments in structure determination problems.

He then detailed some specific case studies of published applications of the restrained simulation technique, beginning with a 2004 JACS paper [15] using data from site-directed spin-labelling of acyl co-enzyme A binding protein (ACBP) to investigate the residual structure present in the unfolded protein. Restraints were imposed on the average over a set of copies (replicas) of the molecule and the technique was implemented through 25 different non-interacting models of the molecule — multiple simulations increased the accuracy of the back-calculation of non-restrained values. Not all of several hundred possible restraints are used in any given model, but those used have to be mutually consistent.

Vendruscolo showed contact maps of the native and denatured states, maps of the average distances between pairs of residues (these were, in fact, based on the probabilities of the interactions between pairs of residues). Although denatured ACBP molecules are highly heterogeneous, Vendruscolo claimed that the sensitivity of the computational technique allowed him and his co-workers to identify long-range conformational tendencies.

He also gave other example applications: the identification of rare (e.g. once a day) but large structural fluctuations from the native state [26], based on hydrogen exchange with solvent; the investigation of transition states too short-lived to be investigated properly experimentally; and the modelling of amyloid fibres using solid-state NMR-derived distance restraints.

**José Saldanha** (**http://mathbio.nimr.mrc.ac.uk/taylor/members/jsaldan/**) of Willie Taylor's lab then led us through a rich case history of the application of comparative modelling to the analysis of a therapeutic target molecule. Although a useful technique, comparative modelling can be difficult to present scientifically because its application rarely makes a good 'story'. It is often a step in a larger process or a door to a wider biological question. Saldanha had worked in collaboration with Daruka Mahadevan, a consultant oncologist at the University of Arizona. Saldanha did bioinformatics to analyse targets proposed by his collaborator; Mahadevan performed expression studies.

Saldanha first provided some background on prostate cancer, the second most common form of death in males, and on prostate-specific membrane antigen (PSMA), the main target for his investigations, giving reasons why it might well be a better marker for prostate disease than the widely-known prostate-specific antigen (PSA). PSMA is a 750 amino acid protein, implicated in many body functions — questions were later asked about the wisdom of choosing such a widely-used target. Saldanha's choice rested on several bases: there are several isoforms of PSMA, and the form expressed in prostate cancer is distinct from the others; tumour endothelial cells express it, but not normal endothelial cells; and other researchers are targeting PSMA in prostate cancer. There is also good clinical evidence from early trials that PSMA can be manipulated specifically and safely.

Saldanha ran through the range of bioinformatics programs that were applied to the problem, including BLAST (sequence search), PSIPRED (secondary structure prediction), THREADER (fold recognition), SAP (a structure-based sequence

Copyright © 2004 John Wiley & Sons, Ltd. 

alignment program) and QUANTA (a commercial modelling suite). This process of bioinformatic characterization ran from determining domain boundaries to alignment to structure prediction. It turned out that the transferrin receptor was likely to be the best template. Although distantly related to PSMA, it has a similar domain structure. The two molecules may share similar properties of dimerization and a similar binding–recycling model.

Saldanha's model(s) proved consistent with mutagenesis data and suggested an apical domain that might be involved in substrate binding. Docking of the natural dipeptide substrate, NAAG, hinted that the specificity pocket might be distinctive enough to help in the design of inhibitors, but a full 3-D structure is yet to be experimentally determined.

Workers in Janet Thornton's large group at the European Bioinformatics Institute (EBI) have been seeking to infer function from structural information for some time now. **James Watson** (**http://www.ebi.ac.uk/Information/Staff/person_maint.php?person_id=345**) outlined their efforts to obtain functional assignments within structural genomics work, particularly in collaboration with the Midwest Center for Structural Genomics (**http://www.mcsg.anl.gov/**).

Watson pointed out that, when it works, functional assignment from three-dimensional structure is more appropriate to the identification of biochemical rather than biological function. Currently sequence methods are the most successful way to assign function, but structure-based methods can provide additional functional information. There are still plenty of occasions when no bioinformatic methods work and function can only be identified by direct experiment.

Watson described ProFunc, a bioinformatics pipeline combining a variety of methods [13]. The structural contributions come from matching homologous folds, a variety of 3-D template methods, binding site identification and structure motif (for example helix–turn–helix) conservation. Databases of 3-D templates describe enzyme active sites, ligand binding sites and DNA binding sites. Hits to these templates are ranked by comparing the surrounding environment of the match and calculating a similarity score. He also described the use of 'nests', small structural motifs involving protein backbones that are commonly found to stabilize some secondary structures and can also stabilize ligand binding. The structural alignments come from firstly centring on the 3-D template match (e.g. enzyme active site) then expanding the alignment based on sections considered 'fittable' (within an RMSD cut-off) that consist of at least seven consecutive residues.

Sadly, I was only able to catch the end of **David Burke**'s (**http://www-cryst.bioc.cam.ac.uk/~dave/**) presentation, '*Ab initio* structure prediction' [2,4], and the subsequent discussion. When I arrived, Burke was addressing the question of how to filter tens of thousands of models of loops. Currently, van der Waals' overlap was the main criterion, but he suggested that molecular dynamics force fields, solvent accessibility and comparison with known structures could all be applied to winnow the output from modelling programs. Burke also summarized the questions that still concerned him — and concern many structural bioinformaticians:

- Is it best to separate the selection of the models from the generation of models?
- Has the majority of the reasonable peptide conformations in the protein universe been observed in the structures deposited in the PDB to date?
- How can distantly related molecules be modelled?

Many of us had heard Willie Taylor (**http://mathbio.nimr.mrc.ac.uk/taylor/members/wtaylor/**) talk before, but he promised us that 'Folds, knots and tangles' would include both 'something old and something new' amongst a collection of methods which, although apparently disconnected, all could contribute towards *ab initio* structure prediction. He began by describing the universe of non-redundant folds by type ($\alpha$, $\alpha\beta$ and $\beta$) and pointed out that this division of foldspace, while superficially illuminating, says less about deep similarities between fold classes, than about how we look at proteins.

Now that Taylor and his co-workers are actively interested in model 'proteins' (i.e. non-biological structures devised *in silico*), he has found that they are difficult to classify by eye and they have used Ptitisyn and Finkelstein's concept of *structural layers* to find a way to compare them without the perennial problems of using, say, RMS deviations between $\alpha$ and $\beta$ proteins.

Copyright © 2004 John Wiley & Sons, Ltd. 

Taylor's talks benefit from being supported by live demos of actual programs running on a Linux laptop, rather than static computer slides. He first used RasMol to show the cell matrices he plots from the distribution of his fold types along axes of complexity, and 'curl and stagger'. He has described this classification and its sub-classifications as a 'Periodic Table' of protein structures [25]. In his demonstration this representation was completely dynamic, with individual spheres being clickable to give the SAP representation of each protein fold's superimposed structures — colour-coded by their strength of mutual correspondence [23].

He now uses this scheme for the classification of model proteins. When asked about the RasMol renderings of such elements, Taylor pointed out that these projections represent the architecture of the protein, failing to discriminate, for example, between parallel and anti-parallel $\beta$-strands, but the full topology for each protein is recorded in a 'topology-string' and can be used if needed [11]. Taylor then moved on to questions of *ab initio* protein structure prediction and contrasted his whole-structure interests with the loop-focused work of David Burke, who had preceded him.

Taylor used a constrained random walk to generate structures, along the way occasionally generating secondary structure elements — sometimes domains. A random walk combined with a system for the generation of layers produces structures which are more protein-like. Occasionally this approach results in the production of knots. This behaviour had to be suppressed with 'smoothing'. Some real proteins in the PDB could not be smoothed down to a line. It turned out that these special cases are *knotted*. This curious, almost-incidental discovery led to a publication in *Nature* [24].

Smoothing can be used to compare the complexity of proteins. According to the number of self-hits of smoothed proteins, TIM barrels are simpler than Rossman folds, for example. It is possible to grow protein traces *in silico* through the building of local contacts and plot the ease of building a given fold making only local connections from a specified point in its structure.

Finally, Taylor ran through some of the elements used in his *ab initio* folding experiments:

- Secondary structure predicted with PSI-PRED.
- Random walks generated with RAMBLE.
- Filtering performed using radius of gyration.
- Filtering for knots.
- Filtering for complexity.
- Folds scored (of the order of $10^5$ in number) with CAO (Contact Accepted MutatiOn) [14].
- POPS (the solvent accessibility algorithm described by Fraternali) and SPREK.

Alternative structures produced using his group's *ab initio* methods can be ranked in order by fold and clustered. He hoped to have a comprehensive system using these or similar techniques up and running in time for the next CASP meeting.

Another local speaker, **Vijayalakshmi Chelliah** of Cambridge University's Biochemistry Department, moved us on from protein structure determination to protein function determination with her talk on 'The identification of interacting sites in protein families'. She started from the reasonable premise that critically important residues tend to be conserved by the members of protein families. She had used HOMSTRAD to generate 96 environment-specific substitution tables for protein residues and taken these as a background against which to detect important sites, those where residues are more conserved in families than would be expected from the tables.

The method is simple and logical:

- Make a structure-based alignment of family members.
- Compare the observed and expected substitution patterns.
- Measure the informational difference between the two.

The higher the score, the more distant the two distributions are. High-scoring positions identified in this way are those considered most likely to be functional. These scores can then be mapped onto structures to find high-scoring clusters. For this last stage, Chelliah used Kin3Dcont, part of the kincontour program (**http://kinemage.biochem.duke.edu/index.php**) produced by the Richardson Lab at Duke University, North Carolina.

Chelliah was careful to ignore large gaps when making alignments and to restrict her analysis to sequences with less than 80% mutual identity in order to minimize the noise from 'briefly' conserved, but functionally unimportant, residues.

Copyright © 2004 John Wiley & Sons, Ltd. 

In most of around 250 families the ‘averaged out’ active site predicted was between 0 Å and 9 Å from the true active site, but the method missed functional sites that were indirectly involved in the activity of proteins and sites that were buried. Along the way to these results she made some interesting observations:

- Critical residues that were also structurally important did not score as highly as might have been expected by this method.
- Even inaccessible residues turned out to be very highly conserved — Chelliah put this down to their being important to the structural integrity of active sites in the molecule.
- She felt that this might have been countered by looking for sites retained in both orthologues and paralogues and tested this by adding in phylogenetic information. As it turned out, the addition of close homologues generated more noise.

She observed, as people often do with methods like this, that the predictions were best when residues were in truly equivalent positions within similar structures.

Returning to structure prediction, ‘Conformational sampling for protein structure determination and prediction’ was the title of **Mark DePristo**’s talk. DePristo is another member of Cambridge’s Biochemistry Department (**http://raven.bioc.cam.ac.uk/~mdepristo/**). He described a method developed (and now used) to check protein models, but which turns out to have a range of useful structure-related applications. He introduced his hybrid approach by summarizing the problem in a series of simple figures. If the solution of a protein structure is a global minimum on an energy (or other scoring function) landscape, then our aim should be to smooth out that landscape to avoid local minima and sample enough of it to find the true minimum. Since there is no definitive solution, we must carefully choose heuristics. DePristo explained the advantages of molecular dynamics/simulated annealing approaches over conjugate gradient/steepest descent ones.

His framework for such investigations, RAPPER, avoids optimizing a non-linear function. Instead it chooses many starting points and applies local minimum-finding methods. Once a general class of structures has been specified, the potential energies of those structures can be compared. Because small deviations from ideal geometry are allowed in the real world and flexibility comes at computational cost, RAPPER fixes many parameters (bond lengths, angles) and samples residue-specific propensity tables and hand-curated conformation libraries. The algorithm constructs reasonable 3-D models consistent with prior structural constraints and additional arbitrary ones, and progresses from the N- to C-terminus of a structure, pruning additions in the wrong conformation.

RAPPER has been applied to loop modelling [2], (re)construction of native ensembles [7], comparative modelling, and crystallographic model generation [8]. More details of the program and its variants are available from the RAPPER Website: **http://raven.bioc.cam.ac.uk/index.php**

**David Jones** (Department of Computer Science, Bioinformatics Unit, University College London, **http://www.cs.ucl.ac.uk/staff/D.Jones/index.html**) spoke on the ‘Detection of native disorder in proteins’. To begin, he joked about the irony of his spending years trying to predict structure from sequence before trying to predict ‘non-structure’ from sequence. He also graciously credited Jon Ward (**http://www.cs.ucl.ac.uk/staff/J.Ward/**) with having done most of the work. After running through the basic assumptions of sequence–structure interdependency, he discussed the various kinds of disordered proteins that were known to exist. Some proteins are partially or completely unfolded yet remain functional, and we assume that this is because their molecules form an ensemble of states, rather than a unique structure. These disordered states could be compact or extended molten globules or random coils and, interestingly, can fold fully on binding.

Jones talked about the blurry line between true disorder and experimental uncertainty in determining protein structures as well as the experimental methods that can be used to detect disorder. He proposed functional classes of disordered regions in proteins: ‘springs and linkers’, modification sites, regions important to the timing of complex assembly, and molecular recognition sites. Functional importance is often assumed to correlate with evolutionary conservation and the work on predicting disorder seems to produce results consistent with this. He also outlined some previous work to identify signals of disorder in proteins.

Ward and Jones had trained a support vector machine (SVM) on a non-redundant set of

Copyright © 2004 John Wiley & Sons, Ltd.

crystal structures and found that they could use it to identify 40% of disordered residues with a 1% error rate. The performance was better for longer regions — over 30 amino acid residues in length — for which the detection fraction and error rates were 80% and 0.1%, respectively. The SVM was then applied across genomes and detection rates compared with biological function (as assigned by gene ontology classifications) [27]. He believed other workers' predictions of disorder in prokaryotic proteins were likely to be overestimates. In eukaryotes, molecules associated with the actin cytoskeleton scored highly, while the bacteria-like environment of mitochondria seemed to contain few disordered protein components. There was also high correlation with DNA-transposition and development and morphogenesis. Molecular functions more likely to be associated with protein disorder predictions included transcription regulators, protein kinases and transcription factors. Metabolic and biosynthetic protein functions scored low. The disorder prediction server, DISOPRED, is available at **http://bioinf.cs.ucl.ac.uk/disopred/disopred.html**

Another Chothia group member, **Martin Madera** (**http://stash.mrc-lmb.cam.ac.uk/mm238/**) talked about his work on 'Comparisons of sequence families' and his responsibility for the Chothia group's 'Superfamily' database at the LMB [16]. This is a library of HMM models for all proteins of known 3-D structure. He recounted a history of protein sequence comparison methods, of the problems of characterizing more distantly related protein groupings, and he detailed more recent improvements in this resource. He gave a clear overview of pairwise vs. sequence profile vs. HMM methods and, having made the case for HMMs, he discussed the refinements implemented in Superfamily, which relies on the segmentation of PDB structures into domains and the combination of multiple HMMs to represent its groupings. The domain-based analysis of Superfamily can now be used to compare whole genomes for their domain composition.

We moved from better models of real, stable, folded proteins, to predictions of disordered proteins to completely imaginary proteins. **Benjamin Blackburne** (**http://slater.chem.nott.ac.uk/∼bpb/**), formerly of Jonathan Hirst's group at Nottingham University and now a member of Richard Goldstein's group, talked about the properties of his phylogenies of minimalist proteins [3]. Blackburne had explored the relationships between hypothetical 2-D proteins catalogued in the sort of protein database the inhabitants of 'Flatland' [1] might recognize. In Blackburne's planar protein universe, residues are of only two types, hydrophobic or hydrophilic. Proteins fold when strings of such residues arranged on a square or tetrahedral lattice of available points turn in on themselves in a plausible way. Folds that arrange those residues with the lowest energy are 'native'. A 'fit' protein is one which has a pocket — i.e. two external residues around a hole that could be 'functional'.

With so few degrees of freedom, all sequences of given short lengths and all structures derived from them can be known. The proteins can be arranged in families, where a family is a group in which all the possible relatives can be generated from another by mutation and yet still meet the rules for the formation of viable structures; the relationships between the model structures can be visualized in graphs, whose nodes are the structures and whose edges are point mutations between them. There are outliers, and some families are more weakly connected to related families than others. There are 'bottlenecks' where there are few evolutionary routes from one family to another. 'Hubs' bridge multiple families. 'Funnels' form when the structures are arranged such that the nodes radiate out to variants of decreasing stability.

Some phenomena can be compared in an illuminating way with the evolution of real proteins. For example, in Blackburne's world neutral evolution seems necessary for minimal proteins to reach functional states and longer chains offer more potential for such noisy change. Other characteristics of these artificial proteins are more problematic: their sequences are not directional and insertions and deletions cannot have the same meaning when there are so few residue types.

The subsequent discussion addressed the relevance of such evolutionary landscapes to real proteins, whether the graphs had scale-free properties, other aspects of real protein behaviour which ought to be modelled (Cyrus Chothia), and the correspondence between Blackburne's neutral mutation-tolerant proteins and Chothia's stable-to-mutation proteins (Willie Taylor).

**Richard Goldstein's** (**http://mathbio.nimr.mrc.ac.uk/goldstein/members/rgoldstein/**) talk, 'Modelling molecular evolution', covered an area

Copyright © 2004 John Wiley & Sons, Ltd. 

of growing interest, the effort to combine sequence and structural analysis to investigate the evolution and function of proteins. He described methods aimed at increasing our understanding of the structural basis for variations in amino acid residue substitution rates, identifying functional sites and, in particular, for characterizing members of the large and pharmacologically important family of G protein-coupled receptors (GPCRs).

First, he highlighted a central flaw in comparative sequence analysis: most approaches are based on a model that assumes positions in sequences represent independent samplings from all possible sequences and ignore the phylogenetic relationships between related proteins. He also reminded us — as molecular phylogeneticists often have to remind biochemists and molecular biologists — that residues 'conserved' between closely related sequences are not as significant as investigators often believe.

Rather than ignore these problems or devise *ad hoc* fixes, Goldstein, Goldman and others have more recently attempted to model evolution explicitly. To begin, Goldstein developed substitution matrices for different types of local structure, but has since devised a more general approach. Each protein can be divided up into zones, without making assumptions about which models apply where; the probability of any given location belonging to a particular site class is a parameter which is itself optimized by an expectation maximization algorithm.

Once a set of environment categories has emerged, Goldstein and co-workers assign qualitative labels to them (e.g. 'hydrophilic'), and the *a posteriori* probabilities of each position belonging to class can be estimated. By applying this approach to large enough families of aligned sequences with structural information, he claimed, it is possible to identify locations where different types of selective pressure have been operating and obtain insights into the underlying basis of such selective pressure, e.g. how physicochemical properties such as size and hydrophobicity are differentially important in different classes of site.

This approach can be used to identify functionally important locations — sites belonging to the slowest evolving rate classes — and different overall probabilities that a position is involved in general function, stabilization, dimerization, packing, structure, or the extent to which a position constrained [21].

Goldstein then focused on the application of this general approach to the specific question of the GPCRs. Despite representing only 1% of the genome, they are estimated to be the target of almost half all drugs and only one signalling process does not involve a member of this family. Although only one known high-resolution structure is available, Goldstein's group worked with a dataset of about 200 GPCRs, and analysed them to produce patchworks of model assignments along the lengths of sequences.

Some properties of these molecules gave a strong signal. It is harder, for example, to identify the inner and outer surface of transmembrane (TM) helices, such as those in the 7-TM structure of the GPCRs, than it is to identify the inner and outer faces of 'normal' protein structure helices. Goldstein *et al.*'s site classes correlate with the 'innerness' and 'outerness' of these helices. Also, a propensity to involvement in dimerization seems to correlate with slowly varying sites.

The European Bioinformatics Institute's **Hugh Shanahan** (**http://www.biochem.ucl.ac.uk/~shanahan/**) described more function-from-structure work, this time targeted at predicting DNA-binding proteins from 3-D motifs and electrostatic information. There is no shortage of important DNA-binding proteins and a huge and growing interest in the regulation of transcription. Shanahan quoted estimates of up to 7% of eukaryotic and 3% of prokaryotic genes coding for DNA binding proteins. Equally, structural genomics projects will generate many uncharacterized structures. Although he acknowledged the importance and utility of sequence-based approaches, he argued that function varies significantly as sequence identity between unknown and known (template) protein sequences falls below 40%. He pointed out that, although at least one neural net-based method exists for identifying DNA binding proteins, it has a high false-positive rate and requires high-resolution atomic data, and claimed that homology-based modelling produces lower false-positive scores.

Shanahan further contended that, of the four main known classes of structural motif:

- Helix–turn–helix.
- Helix–hairpin–helix.

Copyright © 2004 John Wiley & Sons, Ltd. 

- Helix–loop–helix.
- Zinc finger.

the middle two are more easily identified with Hidden Markov Model (HMM) methods; zinc finger proteins are too structurally variable. Shanahan concentrated on the first, helix–turn–helix (H–T–H) structures. He began by summarizing the procedure to identify structural templates:

- Search the literature for H-T-H motifs.
- Identify HMMs in Pfam or SMART.
- Identify structural templates from domains using the CATH super-structural family (the H-level of that database).
- Scan the Protein DataBank with templates.
- Add any new H–T–H DNA-binding proteins to the list.
- Repeat until no other structures are found.

The group obtained 90 non-redundant structures in the PDB and generated seven structural templates to cover that set, applying an accessibility criterion. At first the results didn't seem much better than those obtained with HMMs: 0.5% false positives. Then they refined the method by integrating the potential over a region close to the accessible surface of motifs and tested this by using the electrostatic data to attempt to identify the binding region in known DNA-binding proteins [12].

A method to detect DNA-binding sites on the surface of a protein structure is important for functional annotation. They analysed residue patches on the surface of DNA-binding proteins and predicted DNA-binding sites using a single feature of these surface patches. They first surveyed surface patches and DNA-binding sites for accessibility, electrostatic potential, residue propensity, hydrophobicity and residue conservation. From this, they observed that the DNA-binding sites usually fell in the top 10% of patches with the largest positive electrostatic scores. This knowledge led to their development of a prediction method in which patches of surface residues were selected such that they excluded residues with negative electrostatic scores.

They used this method to make predictions for a dataset of 56 non-homologous DNA-binding proteins and identified 68% of the dataset correctly. Using this data, they improved the false-positive score to 0.02%. Shanahan added that the hybrid method involves fewer parameters than sequence homology, might in future not require full electrostatic calculations to be performed and that it might be possible to use data from homology models to provide a cross-check for HMM searches.

The final talk of the meeting rounded the event off perfectly. **Chris Calladine** (**http://www-civ.eng.cam.ac.uk/crc/crc_web.htm**), who retired only a couple of years ago from the Cambridge University Department of Structural Engineering, dazzled us with a multidisciplinary, multimedia presentation on the 'Mechanics of interfaces in $\alpha$-helical supercoils'. He used overheads, animation and a succession of cork-and-cardboard models to show how juxtaposed helices could abut in diverse ways, interlocking the 'knobs' of their respective sidechains. The knobs of one helix fit into the 'holes' between the knobs of the other when they pack. For simple superhelices and four-helix bundles — as distinct from the helix-built cylinders Calladine later touched on [5] — there were three standard modes of knobs-into-holes packing, which he illustrated with overlaid interface figures produced as overheads, as simple figures and as cleverly constructed three-dimensional models.

One of the most pleasing things about structural bioinformatics is that its practitioners collaborate across specialisms to tackle difficult, interesting and messy problems out of both curiosity and necessity — not merely to meet the conditions of interdisciplinary funding programmes. Calladine's work exemplified this beautifully. He has worked in this area in collaboration with Charlie Laughton (molecular dynamics) at Nottingham University and Ben Luisi and Venkatash Pratap (structural biology) at Cambridge. Pratap wrote software that finds $\alpha$-helices and their neighbours, identifies the local superhelical angle of their arrangement and categorizes those arrangements according to those angles. Pratap's animation of a bistable 'switch' in the packing of a right-handed, four-helix bundle of $\alpha$-helices in one of the three main classes of arrangement formed the finale of Calladine's presentation.

### Acknowledgements

I would like to thank the speakers-especially Franca Fraternali- for their contributions, clarifications, and corrections to this article.

Copyright © 2004 John Wiley & Sons, Ltd. 

### References

1. Abbott EA. 1884. *Flatland: A Romance of Many Dimensions*. Shambhala: Boston, MA.
2. de Bakker PI, DePristo MA, Burke DF, Blundell TL. 2003. *Ab initio* construction of polypeptide fragments: accuracy of loop decoy discrimination by an all-atom statistical potential and the AMBER force field with the Generalized Born solvation model. *Proteins* **51**: 21–40.
3. Blackburne BP, Hirst JD. 2001. Evolution of functional model proteins. *J Chem Phys* **115**: 1935–1942.
4. Burke DF, Deane CM. 2001. Improved protein loop prediction from sequence alone. *Protein Eng* **14**: 473–478.
5. Calladine CR, Sharff A, Luisi BF. 2001. How to untwist an $\alpha$-helix: structural principles of an $\alpha$-helical barrel. *J Mol Biol* **305**: 603–618.
6. Cavallo L, Kleinjung J, Fraternali F. 2003. POPS: a fast algorithm for solvent accessible surface areas at atomic and residue level. *Nucleic Acids Res* **31**: 3364–3366.
7. DePristo MA, de Bakker PIW, Lovell SC, Blundell TL. 2002. *Ab initio* construction of polypeptide fragments: efficient generation of accurate, representative ensembles. *Proteins Struct Funct Genet* **51**: 41–55.
8. DePristo MA, de Bakker PIW, Lovell SC, Blundell TL. 2004. Heterogeneity and inaccuracy in protein structures solved by X-ray crystallography. *Structure* **12**: 831–838.
9. Fernandez-Recio J, Totrov M, Abagyan R. 2004. Identification of protein–protein interaction sites from docking energy landscapes. *J Mol Biol* **335**: 843–865.
10. Fraternali F, Cavallo L. 2002. Parameter optimized surfaces (POPS): analysis of key interactions and conformational changes in the ribosome. *Nucleic Acids Res* **30**: 2950–2960.
11. Johannissen LO, Taylor WR. 2004. Protein fold comparison by the alignment of topological strings. *Protein Eng* **16**: 949–955.
12. Jones S, Shanahan HP, Berman HM, Thornton JM. 2003. Using electrostatic potentials to predict DNA-binding sites on DNA-binding proteins. *Nucleic Acids Res* **31**: 7189–7198.
13. Laskowski RA, Watson JD, Thornton JM. 2003. From protein structure to biochemical function? *J Struct Funct Genomics* **4**: 167–177.
14. Lin K, Kleinjung J, Taylor WR, Heringa J. 2003. Testing homology with Contact Accepted mutatiOn (CAO): a contact-based Markov model of protein evolution. *Comput Biol Chem* **27**: 93–102.
15. Lindorff-Larsen K, Kristjansdottir S, Teilum K, *et al.* 2004. Determination of an ensemble of structures representing the denatured state of ACBP. *J Am Chem Soc* **126**: 3291–3299.
16. Madera M, Vogel C, Kummerfeld SK, Chothia C, Gough J. 2004. The SUPERFAMILY database in 2004: additions and improvements. *Nucleic Acids Res* **32**: (Database issue): D235–239.
17. Mizuguchi K. 2004. Fold recognition for drug discovery. *Drug Discovery Today: Targets* **3**: 18–23.
18. Shi J, Blundell TL, Mizuguchi K. 2001. FUGUE: sequence–structure homology recognition using environment-specific substitution tables and structure-dependent gap penalties. *J Mol Biol* **310**: 243–257.
19. Shirai H, Blundell TL, Mizuguchi K. 2001. A novel superfamily of enzymes that catalyze the modification of guanidino groups. *Trends Biochem Sci* **26**: 465–468.
20. Shirai H, Mizuguchi K. 2003. Prediction of the structure and function of AstA and AstB, the first two enzymes of the arginine succinyltransferase pathway of arginine catabolism. *FEBS Lett* **555**: 505–510.
21. Soyer OS, Dimmic MW, Neubig RR, Goldstein RA. 2003. Dimerization in aminergic G-protein-coupled receptors: application of a hidden-site class model of evolution. *Biochemistry* **42**: 14 522–14 531.
22. Stebbings LA, Mizuguchi K. 2004. HOMSTRAD: recent developments of the Homologous Protein Structure Alignment Database. *Nucleic Acids Res* **32**: (Database issue): D203–207.
23. Taylor WR. 2000. Protein structure comparison using SAP. *Methods Mol Biol* **416**: 657–660.
24. Taylor WR. 2000. A deeply knotted protein structure and how it might fold. *Nature* **406**: 916–919.
25. Taylor WR. 2002. A ‘periodic table’ for protein structures. *Nature* **416**: 657–660.
26. Vendruscolo M, Paci E, Dobson CM, Karplus M. 2003. Rare fluctuations of native proteins sampled during equilibrium hydrogen exchange. *J Am Chem Soc* **125**: 15 686–15 687.
27. Ward JJ, Sodhi JS, McGuffin LJ, Buxton BF, Jones DT. 2004. Prediction and functional analysis of native disorder in proteins from the three kingdoms of life. *J Mol Biol* **337**: 635–645.

Copyright © 2004 John Wiley & Sons, Ltd. 
